# Experimental study on the impact of “IDS + JFCS” complex wetting agent on the characteristics of coal bodies

**DOI:** 10.1038/s41598-024-57443-x

**Published:** 2024-03-26

**Authors:** Hongyang Wang, Lianman Xu, Zhijiao Qin, Xiaoliang Li, Xuejing Cao, Yumiao Han, Siqi Li, Yufei Ma, Siqi Gao, Lei Du, Fengshuo Yang

**Affiliations:** 1 School of Environment, Liaoning University, Shenyang, 110036 China; 2 Liaoning Qingyang Specialty Chemical Co, Liaoyang, 111000 China

**Keywords:** Impact ground pressure, Coal bed water injection, Surfactant, Complex wetting agent, Environmental sciences, Natural hazards, Solid Earth sciences, Energy science and technology

## Abstract

As China's coal mines have transitioned to deep mining, the ground stress within the coal seams has progressively increased, resulting in reduced permeability and poor wetting ability of conventional wetting agents. Consequently, these agents have become inadequate in fulfilling the requirements for preventing washouts during deep mining operations. In response to the aforementioned challenges, a solution was proposed to address the issues by formulating a composite wetting agent. This composite wetting agent combines a conventional surfactant with a chelating agent called tetrasodium iminodisuccinate (IDS). By conducting a meticulous screening of surfactant monomer solutions, the ideal formulation for the composite wetting agent was determined by combining the monomer surfactant with IDS. Extensive testing, encompassing evaluations of the composite solution's apparent strain, contact angle measurements, and alterations in the oxygenated functional groups on the coal surface, led to the identification of the optimal composition. This composition consisted of IDS serving as the chelating agent and fatty alcohol polyoxyethylene ether (JFCS).Subsequent assessment of the physical and mechanical performance of the coal briquettes treated with the composite wetting agent revealed notable enhancements. These findings signify significant advancements in the field and hold promising implications. Following the application of the composite wetting agent, notable reductions were observed in the dry basis ash and dry basis full sulfur of coal. Additionally, the water content within the coal mass increased significantly, leading to a substantial enhancement in the wetting effect of the coal body. This enhanced wetting effect effectively mitigated the coal body’s inclination towards impact, thereby offering technical support for optimizing water injection into coal seams and preventing as well as treating impact ground pressure.

## Introduction

Coal seam water injection serves a multitude of purposes, encompassing the augmentation of gas extraction rates, mitigation of coal-rock impacts, enhancement of top coal risk alleviation, and prevention of mine dust. It is regarded as a fundamental approach in the prevention and control of power disasters, particularly deep-impact ground pressure^1^. However, in practical coal bed water injection projects, the suboptimal wetting performance of the coal body often results in unsatisfactory outcomes when pure water injection is employed. Moreover, endeavors to enhance the efficiency of water injection into coal seams have encountered limitations. Despite adjustments in water injection parameters and techniques, notable advancements have not been achieved, thus falling short of the prerequisites for effective prevention and control of impact ground pressure. Consequently, in practical coal seam water injection engineering, the integration of surfactants has emerged as a common strategy. These additives aim to augment the coal's hydrophilicity, facilitate unhindered water flow through the coal body's pores, and optimize the overall efficacy of water injection into the coal matrix^2^. However, with the increasing depth of coal mining operations, the coal body undergoes compaction, resulting in a reduction of its porosity. This presents challenges for conventional surfactant solutions, as they may encounter difficulties in adequately wetting the coal body, leading to suboptimal outcomes during the water injection process. Consequently, achieving the desired objective of preventing and controlling impact ground pressure becomes challenging^3^. In the context of high stress and low permeability conditions in deep coal seams, the frequency and severity of impact ground pressure accidents are significantly amplified^4^.

In recent years, numerous research studies have focused on the utilization of chelating agents and surfactants. Sulastri et al. conducted an evaluation of various chelators, including Ethylenediaminetetraacetic acid (EDTA), to assess their adsorption capacities for toxic cooking oil contaminants such as Pb and As. The findings revealed that EDTA and other chelators exhibited effective adsorption of toxic cooking oil, including Pb and As, in soil^5^. Tolkacheva et al. conducted research demonstrating that the chelating agent IDS can form stable complexes through its reaction with Al^3+^^6^. Furthermore, Xu et al. discovered that the anion present in the chelating agent IDS can react with easily precipitated ions in coal, such as Ca^2+^ and Mg^2+^ forming stable cyclic complexes. This reaction prevents the precipitation of these ions with carbonates and impedes the obstruction of coal pores^7^. Sun et al. conducted a study aimed at enhancing the wetting efficiency of water injection in coal seams and mitigating dust generation at the source. They proposed the development of three effective wetting enhancers^8^. Wang et al. conducted triaxial percolation experiments and spontaneous wetting experiments with the objective of expanding the application of water injection in coal seams, improving the effectiveness of water wetting in coal beds, and identifying the suitable ionic surfactants for coal bed water injection^9^. Their research investigated the influence of oxygen-containing functional groups and minerals present in coal on coal's wettability. Additionally, they provided a microscopic perspective to elucidate the wetting mechanism of SDBS and SDS^10^. Laboratory experiments, as well as numerous field experiments, have been conducted to examine the effects of varying concentrations of monomer surfactants and composite surfactants on coal's wettability^11^. Furthermore, extensive research has been dedicated to exploring the impacts of chelating agents on the wetting characteristics of coal, as well as the effects of surfactant monomers and their composite solutions on coal^11^. However, limited research has been conducted on the effects of innovative wetting agents formulated by combining chelating agents and surfactants on the properties of coal bodies.

## Experimental

### Laboratory materials and facilities

#### Laboratory materials

Samples of coal utilized in the experiment were obtained from the designated 703 working faces of the mine. The coal seam present in this specific working face predominantly comprises low-quality coal characterized by a high degree of coalescence. This coal seam is situated at significant burial depths, subjected to concentrated stress, and exhibits limited permeability. Moreover, the coal seam displays a discernible inclination towards experiencing impacts. For the purpose of the experiment, a collection of coal samples of uniform dimensions was procured from the gluing roadway of the 703 working faces. A part of the sample was evenly crushed with a hammer, a part was used for infrared test experiments with a particle size of 0.15 mm, and the other part was used for coal quality analysis with a particle size of 0.075 mm. Then keep it in a sealed bag. The other portion was cut into standard specimens with dimensions of 5 cm × 5 cm × 10 cm and sealed with plastic wrap for preservation.

The chelating agent IDS (referred to as such in the paper) was chosen as a monomeric compound wetting agent due to its capacity to interact with minerals. This interaction facilitates the conversion of insoluble minerals into a soluble state, thereby enhancing the interconnectivity of internal pores within the coal body. Consequently, this process improves the wetting effectiveness of water when it permeates the coal matrix^12^. The researchers opted for IDS as the monomeric compound wetting agent based on its non-toxicity, innocuous nature, and favorable wetting performance. In order to formulate a composite wetting agent with enhanced wetting properties, a combination of chemicals exhibiting superior wetting characteristics was developed. Six surfactants, representing four different types commonly employed in the industry, were selected as potential monomers for compounding. These surfactants, in conjunction with the chelating agent IDS, are listed in Table 1 of the paper, which provides details regarding their fundamental properties.

**Table 1:** Basic properties of complex monomers.

Types of chemical	Abbreviations	Abbreviations	Chemical formula	CAS
Chelating agent	Tetrasodium iminodisuccinate	IDS	C_8_H_7_NO_8_Na_4_	144538-83-0
Anionic surfactants	Dodecyl Sodium Sulfonate	SAS	CH_3_(CH_2_)_11_SO_3_Na	2386-53-0
Anionic surfactants	Sodium dodecylbenzene sulfonate	SDBS	C_18_H_29_NaO_3_S	25155-30-0
Cationic surfactants	Hexadecyltrimethylammonium bromide	1631	C_16_H_33_(CH_3_)_3_NBr	112-02-7
Amphoteric surfactants	Dodecyl betaine	BS-12	C_16_H_33_O_2_N	683-10-3
Nonionic surfactants	Alkyl glycosides	APG	RO (G)n	161074-87-9
Nonionic surfactants	Fatty alcohol polyoxyethylene ether	JFCS	RO(CH_2_CH_2_O)nCH_2_CH_2_OH (R = C_12_-C_18_ Alkyl, n = 5–6)	68439-50-9

#### Laboratory facilities

**Figure 1:**
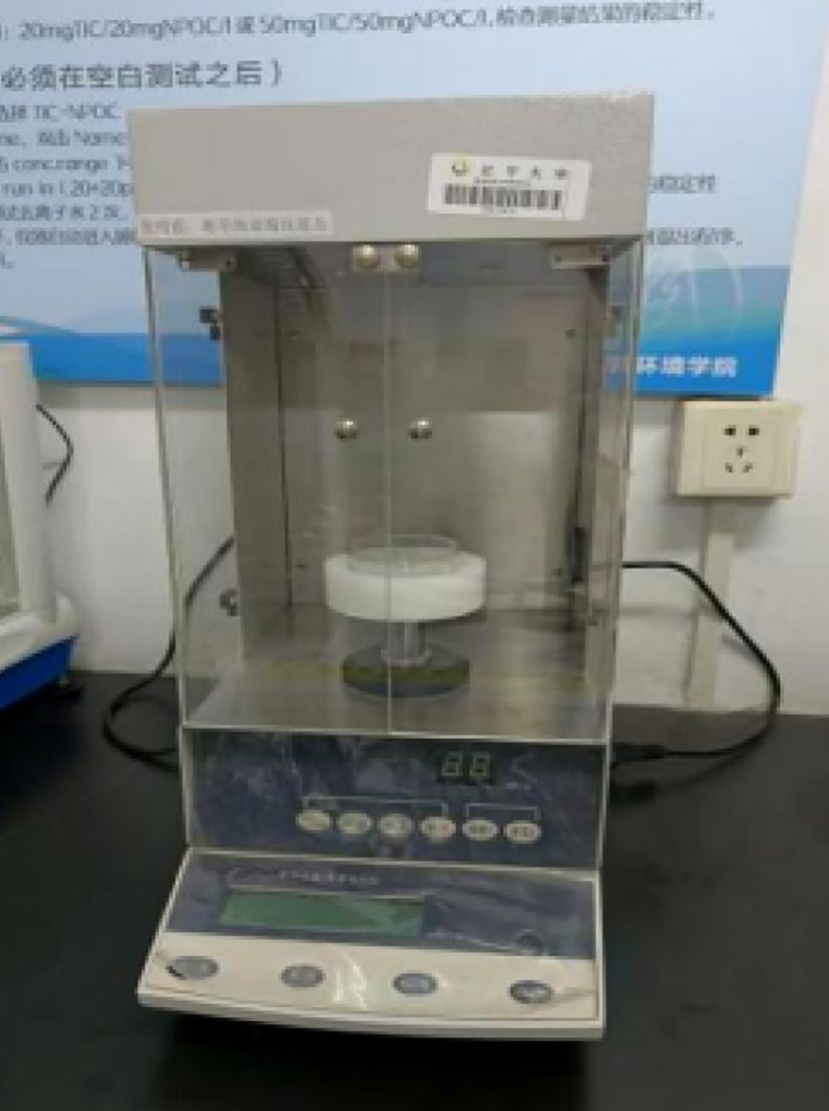
Automatic surface tension meter (BZY-1).

1. Experimental equipment for surface tension and contact angle determination.
  The surface tension of the chelating agent, monomer solution of surfactant, and composite liquid was determined using an automatic surface tension meter (BZY-1). (See Fig. 1). After 30 s of contact between the solution and the coal, test photos were taken using a digital camera (see Fig. 2) to measure the contact angle.

**Figure 2:**
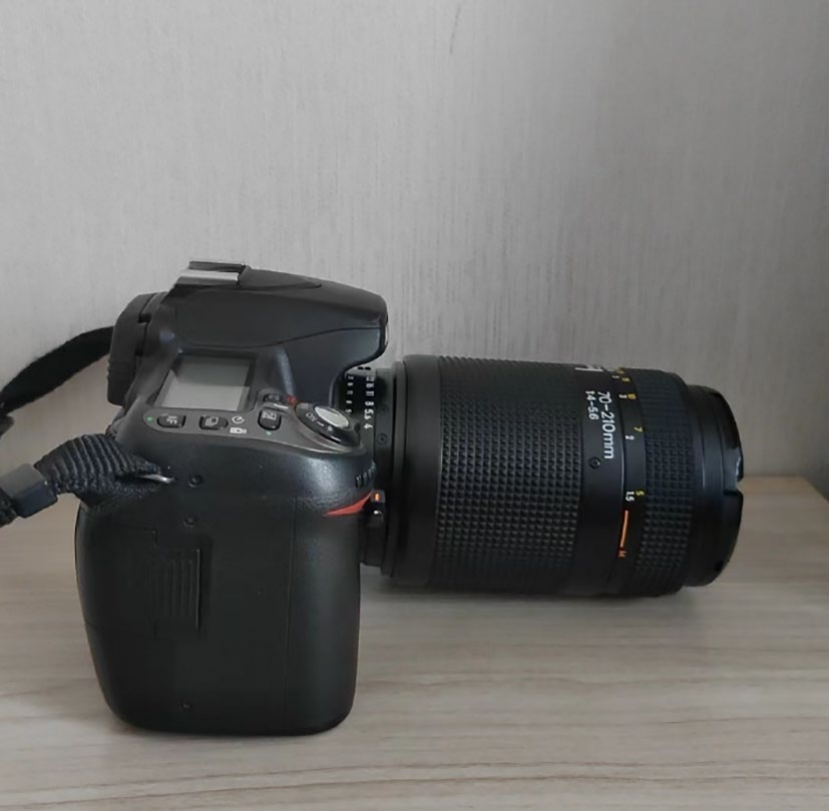
Digital camera.
2. The experimental apparatus employed for the analysis of surface functional groups in coal samples.
  In order to assess the effects of the compound solution on the modification of the coal body, Fourier infrared spectroscopy was employed to analyze the surface functional groups of the coal samples. This analysis was conducted both before and after exposure to the compound solution and its monomer solution, as illustrated in Fig. 5b^12^. The modifications in the functional groups present on the surface of the coal samples were evaluated by examining the position and intensity of the spectral peaks throughout the utilization of the complex wetting agent.
3. The experimental equipment used to measure the moisture content of the coal sample.
  Measuring the moisture content of coals before and after treatment with the composite solution, the samples were subjected to drying in a WGLL-65BE drying box (refer to Fig. 3) until reaching a constant weight. The moisture content of coals was determined by an electronic analytical balance used to measure the mass of the sample during the drying process.

**Figure 3:**
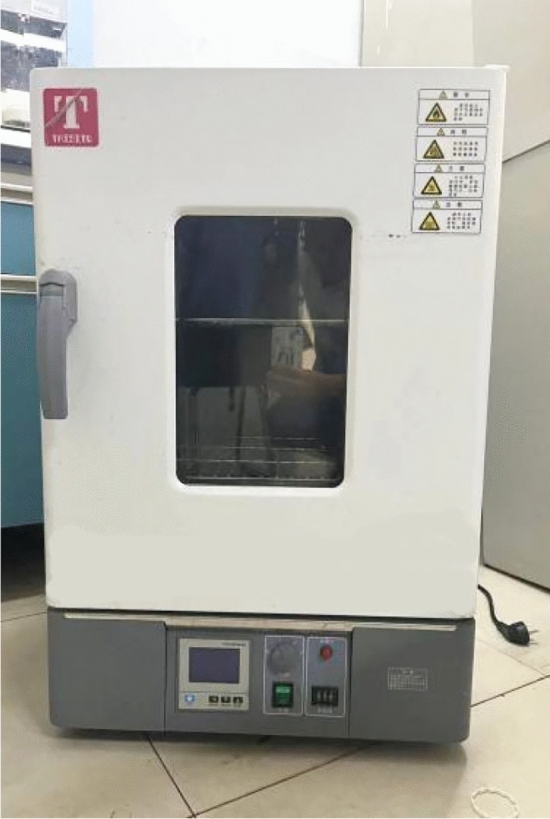
WGLL-65BE drying box.
4. Experimental equipment for determination of coal body physical properties.
  China's current coal impact propensity determination indexes mainly include: the properties evaluated included *σ*_*c*_, *K*_*E*_, *D*_*T*_, and other relevant parameters. To assess the mechanical properties of coal bodies, a 100KN microcomputer-controlled universal testing machine (as shown in Fig. 4) was employed to conduct uniaxial compressive tests on standard coal samples. The obtained data were then analyzed and calculated to generate stress–strain and displacement-load diagrams.

**Figure 4:**
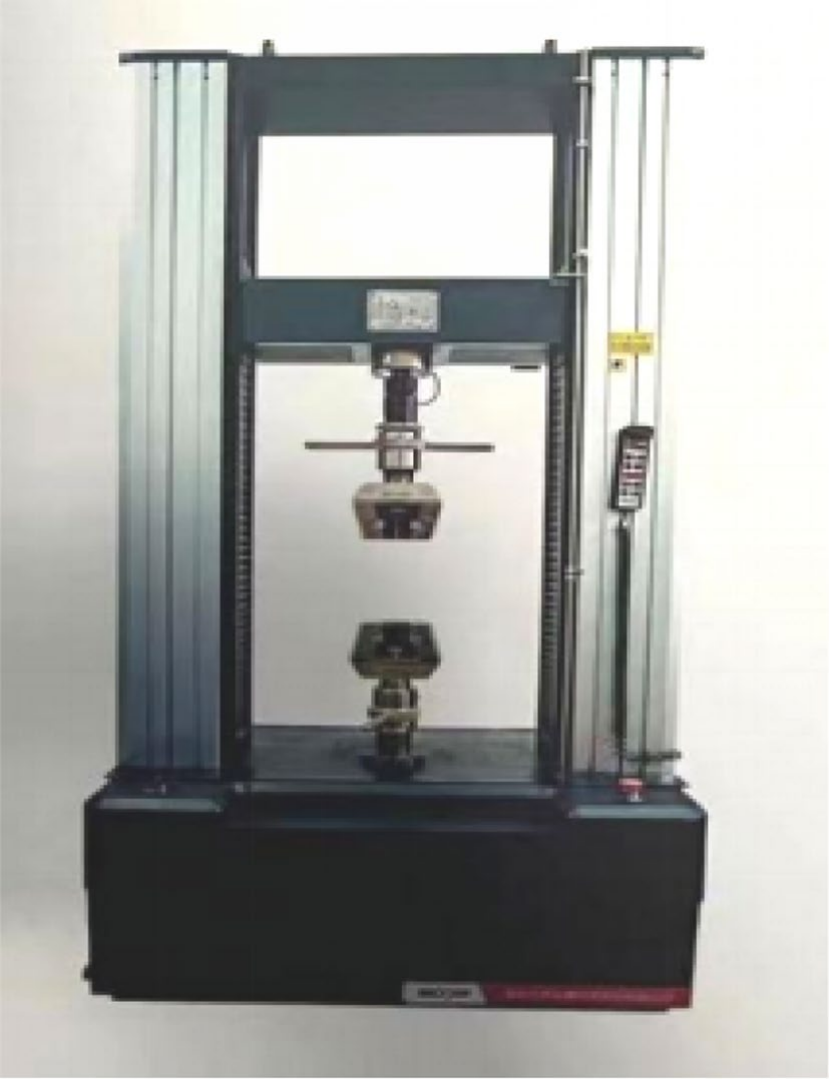
100 KN microcomputer-controlled universal testing machine.

### Experimental process

#### The experimental investigations aimed to ascertain the appropriate composition and concentration of the compound solution

**Figure 5:**
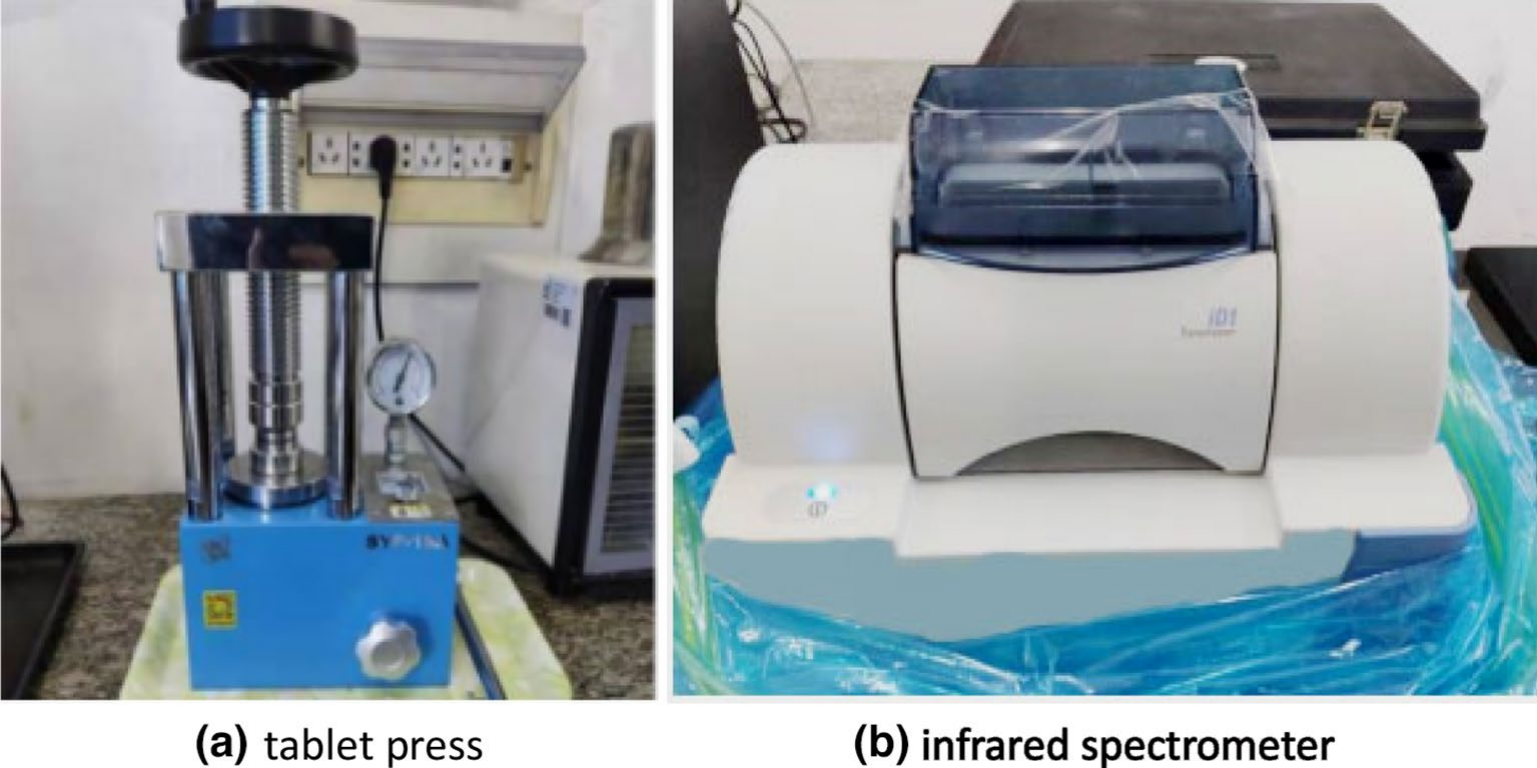
Experimental instruments.

1. Measuring surface tension and contact angle of coal samples.
  IDS with mass concentrations of 0.005%, 0.01%, 0.03%, 0.05%, 0.07%, and 0.09%, and six surfactants SDBS, SAS, 1631, JFCS, BS-12, and APG were prepared and preserved; the different mass concentrations of each reagent were measured three times with a surface tension meter, the final measurement result was obtained by averaging the recorded values. To determine the contact angle, flatten the surface of the coals with sandpaper, and the sanded coal block was then placed on a smooth, flat rock block. A syringe is used to drop the test solution perpendicular to the surface of the coal sample, where the height of the syringe is 5 cm from the sample, and the titration is 0.05 ml per time. The contact angle was measured using a surface tension meter for each reagent solution with varying mass concentrations^13^. The contact angle tests were conducted three times on the surface of the coal body using solutions of different reagents at various mass concentrations. The final determination was obtained by calculating the average value of the measured contact angles. Furthermore, the selected surfactant monomers were individually combined with IDS in pairs. The surface tension and contact angle of the composite solutions were measured following the experimental procedures described earlier.
2. Surface functional group analysis of coal samples.
  For rigorous accuracy in the formulation of compound solutions, further, the surface functional group changes of the coal samples were analyzed. The coal sample is initially crushed through a 5-mesh sieve, and then the screened coal sample is further crushed and then screened through a 100-mesh sieve. Subsequently, 5 g of the coal samples were accurately weighed using an electronic balance. The weighed coal samples were then immersed in the six types of compound solutions and their respective monomer solutions, 1:10 as its solid–liquid ratio, for three days. After the coal samples were soaked, they were carefully rinsed with ultrapure water and subsequently transferred to a drying oven. The samples were subjected to a drying process at a temperature of 105 °C until a consistent weight was obtained. Once completely dried, the coal samples were meticulously blended with dried potassium bromide in a precise ratio of 1:100 within a mortar, ensuring thorough and uniform distribution. Subsequently, the resulting mixture was transferred to a pellet press, where it underwent compression under a pressure range of 1.0–1.5 MPa, skillfully fashioned into circular, thin slices^14^. The tablet press and infrared spectrometer used in the experiment are shown in Fig. 5.

#### Experimental studies were conducted to investigate the effect of complex wetting agents on coal quality

1. Coal quality measurement experiment.
  The assessment of complex wetting agents' impact on coal quality is essential for determining their suitability for practical engineering applications^15^. To evaluate this, the focus was placed on the coal quality before and after immersion in IDS and the complex wetting agent, while considering that ultrapure water and JFCS solely affect the wettability of the coal matrix without influencing its quality. The coal samples were extracted, crushed, and sieved, followed by sealing and preservation. Solutions of IDS (with a mass concentration of 0.05%) and the complex wetting agent were prepared. The coal samples were immersed in the IDS and complex wetting agent solutions for a period of 3 days, with the solution being replaced every 24 h while maintaining the same mass concentration. A solid–liquid ratio of 1:10 (coal sample to solution) was employed. After the immersion, the coal samples underwent a 24-h drying period in a drying oven to assess their coal quality. Subsequently, the coal quality of the samples was determined.
2. Coal body water content measurement experiment.
  The water content of coal serves as a detection method for assessing the effectiveness of water injection in coal seams. By analyzing the variations in water content within the coal body, an initial evaluation of the water injection effect in the coal seam can be conducted^16^. Standard coal samples were carefully sealed using plastic wrap and left undisturbed. Each standard coal sample was appropriately labeled, and the coal samples were immersed in ultrapure water, a complex wetting agent, and two monomer solutions for a soaking period of 7 days. Afterward, the surface moisture on the coal samples was meticulously removed, and an electronic analytical balance was utilized to measure the sample's mass. The dried coal samples were then placed in a drying box with a consistent temperature of 105 ℃ until a constant weight was achieved. Subsequently, the cooled dried coal samples were weighed to determine their mass and calculate the moisture content through the measured coal sample quality.
3. Impact susceptibility testing of coal samples.
  Impact susceptibility is an intrinsic factor that determines whether the coal rock can be impacted by ground pressure, which is not affected by other external factors^16^. The judgment indexes of impact propensity mainly include: Parameters such as the *σ*_*c*_, *K*_*E*_, *D*_*T*_, etc. were evaluated. Its specific determination standard is the standard of Pan et al.^17^. Its determination criteria are shown in Table 2.

**Table 2:** Criteria for determining impact propensity of coal.

Form	Dynamic destruction time (ms)	Impact energy index	Uniaxial compressive strength (Mpa)	Propensity to shock
Category I	D_T_ > 500	*K*_*E*_ < 1.5	*σ*_*c*_ < 7	None
Category II	50 < D_T_ ≤ 500	1.5 ≤ *K*_*E*_ < 5	7 ≤ *σ*_*c*_ ≤ 14	Weak
Category III	D_T_ < 50	*K*_*E*_ ≥ 5	*σ*_*c*_ ≥ 14	Vigorous

**Figure 6:**
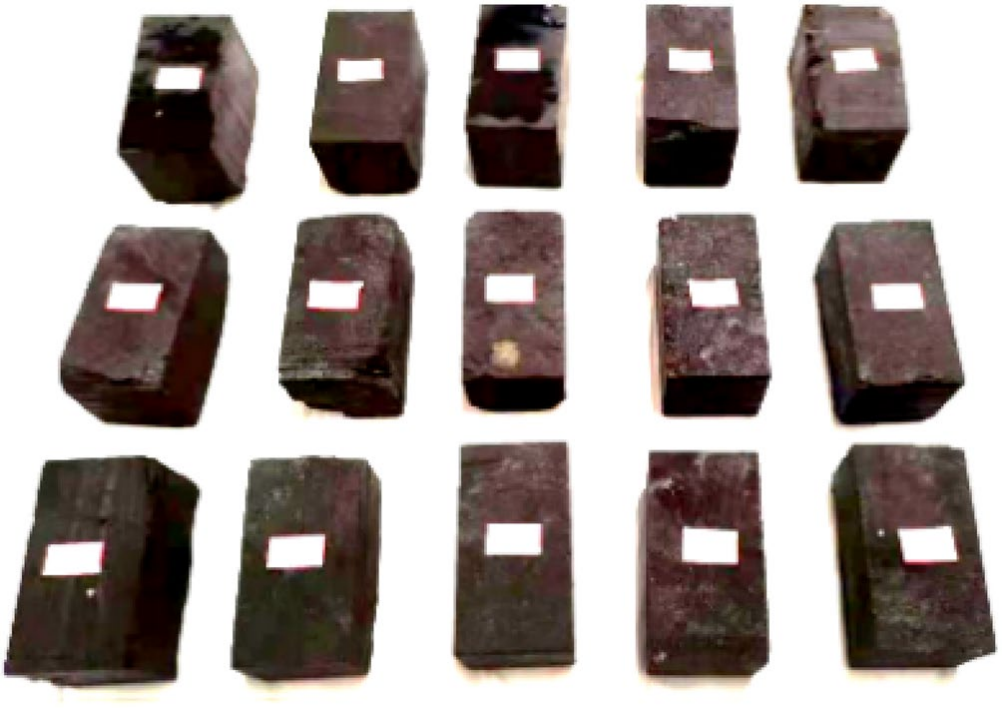
Standard coal sample.

**Figure 7:**
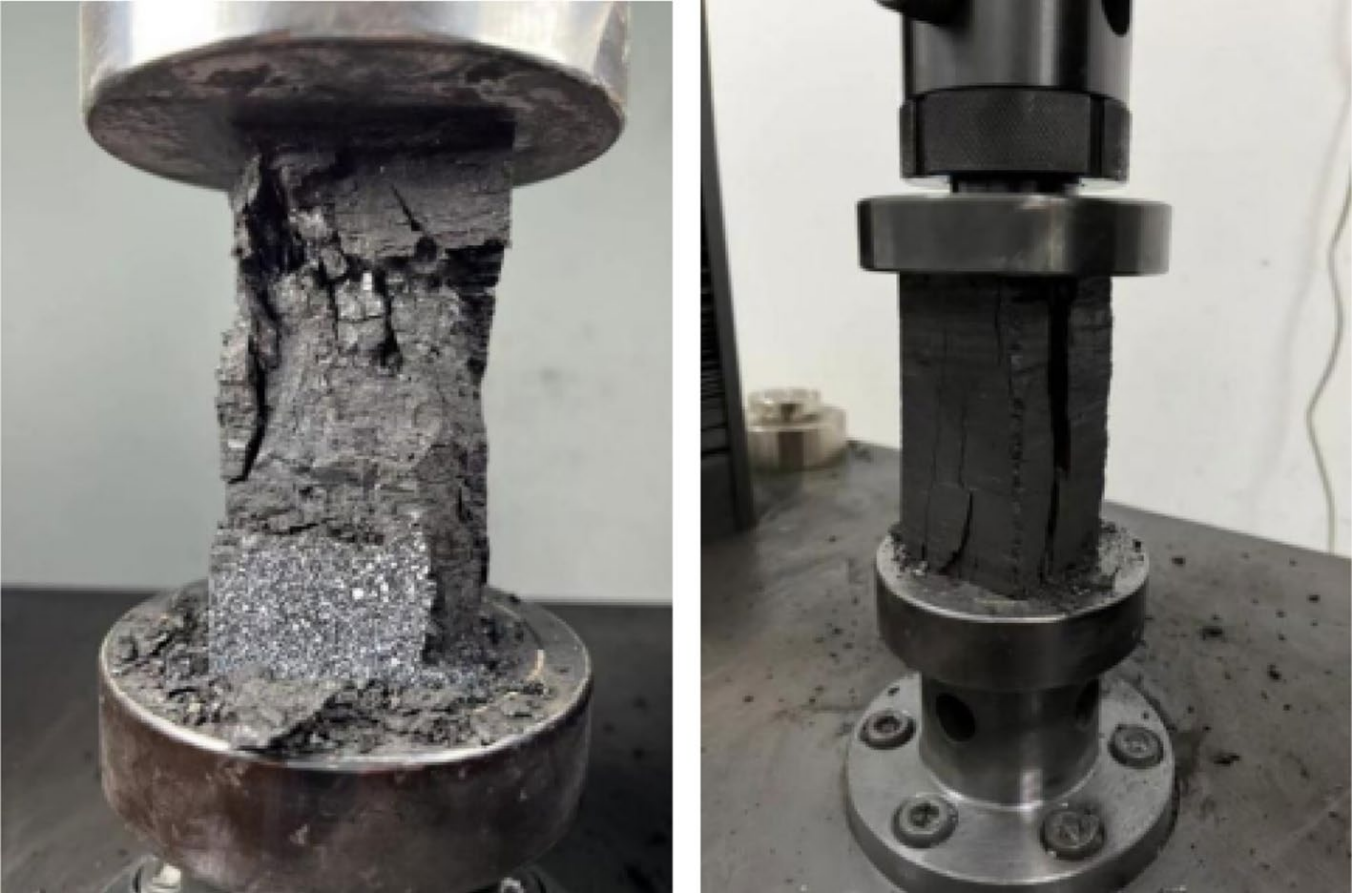
Experiments on the uniaxial compression process of a coal body.

The standard coal samples utilized in the experiment are labeled as depicted in Fig. 6, the standard coal samples were soaked in a complex wetting agent, and its monomer solution for 7 days. Subsequently, the σc underway on the standard coal samples with the 100KN microcomputer-controlled universal testing machine. Before the measurement, the hydraulic testing machine should be made in a workable state, and set the test parameters, respectively. Before and after immersing the standard coal samples, they were positioned in the center of the press bearing board, and the spherical seat was adjusted accordingly so that the standard coal samples on the cross-section of the hydraulic machine and the hydraulic machine on the plane of the platen is parallel to the plane of the platen, Fig. 7 illustrates the experimental procedure^18^.

## Findings and analysis

### Surface tension and contact angle measurement

1. Surface tension measurement results.
  Surface tension is a crucial parameter for evaluating the ability of the wetting agent to moisten the coal body, the lower the surface tension of the solution, the better the moistening effect. Table 3 presents the measurement results of surface tension, while Fig. 8 illustrates the corresponding change curve. (During the experiment, the surface tension of ultrapure water utilized for solution preparation was measured at 72.75 mN/m).

**Table 3:** The surface tension of solutions with different mass concentrations (mN/m).

Name	Mass concentration (%)					
	0.005	0.01	0.03	0.05	0.07	0.09
IDS	72.17	71.73	71.87	71.47	71.80	71.53
SAS	64.23	56.63	53.5	42.87	44.40	42.40
SDBS	72.23	66.8	60.03	53.53	48.73	39.97
1631	20.7	19.30	19.03	16.17	18.37	16.77
JFCS	60.33	58.73	51.07	36.53	41.93	37.40
BS-12	64.57	63.20	56.87	43.60	40.97	34.40
APG	32.33	28.80	28.27	28.23	28.20	28.10

**Figure 8:**
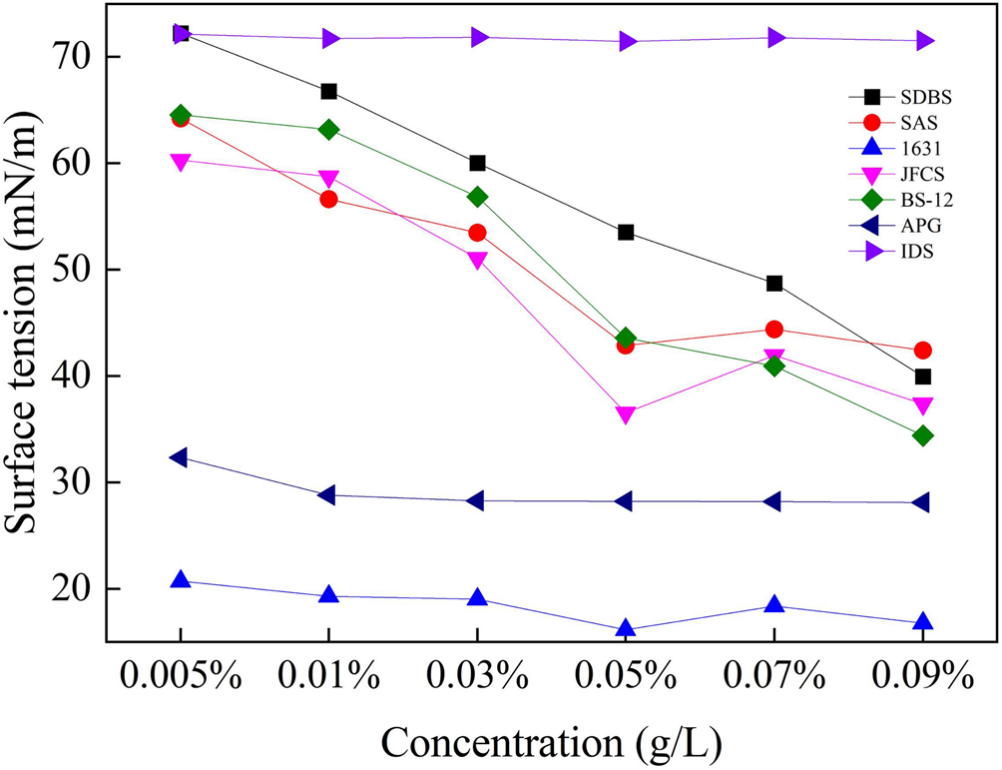
Variation of surface tension with solution mass concentration.
  Based on the findings illustrated in Fig. 8, it is evident that the IDS solution exhibits a higher surface tension, which remains relatively constant regardless of the increase in solution mass concentration. This indicates insufficient wetting ability of the IDS solution towards the coal body. A consistent pattern is observed in the surface tension of the six surfactant solutions. As the solution mass concentration increases, the surface tension decreases. However, the surface tension reaches a plateau when the solution mass concentration reaches 0.05%. This stability can be primarily attributed to the saturation of surfactant sorption at the coal interface as the solution mass concentration increases. The phenomenon arises from the fact that as the concentrations of the solutions are elevated, the surfactant becomes saturated on the coal surface. Consequently, further increases in the solution mass concentration do not significantly impact the change in surface tension. At this point, JFCS, 1631, and APG reach their respective minimum surface tension values of 36.53 mN/m, 16.17 mN/m, and 28.23 mN/m.
2. Findings from contact angle measurements.
  Generally speaking, when the contact Angle is less than 90°, the contact between the measured object and the liquid is wet, indicating that the liquid can be evenly distributed on the surface of the object. When the contact Angle is greater than 90°, the contact between the measured object and the liquid is non-moist, indicating that the liquid cannot be evenly distributed on the surface of the object, forming water droplets or droplets. A smaller contact angle of the solution indicates a more effective wetting effect. Table 4 presents the results of contact angle measurements, while Fig. 8 illustrates the variation of contact angle over the experimental conditions.

**Table 4:** Contact angle of solutions with different mass concentrations (°).

Name	Mass concentration (°)					
	0.005	0.01	0.03	0.05	0.07	0.09
IDS	66	64	60	56	50	49
SAS	93	84	71	51	46	39
SDBS	90	84	78	60	56	44
1631	90	80	64	50	46	42
JFCS	75	58	26	0	0	0
BS-12	93	74	72	50	48	42
APG	76	62	38	25	19	0
  Figure 9 illustrates the reduction in contact angle for different surfactant solutions. The JFCS solution demonstrates the most significant contact angle reduction, reaching an impressive 100% at a solution mass concentration of 0.05%. At this concentration, the contact angle value decreases to 0°, indicating the excellent wetting effectiveness of the JFCS solution. On the other hand, the SDBS surfactant solution exhibits the smallest reduction in contact angle, with only a 37.50% decrease observed. However, the maximum reduction is also observed at the concentration of 0.05%. Similarly, the IDS solution shows the maximum change in contact angle at a mass concentration of 0.05%. At a mass concentration of 0.09%, the remaining five surfactants, except for JFCS and IDS, achieve the maximum reduction in contact angle. Notably, the APG solution demonstrates a remarkable 100% decrease in contact angle, effectively reducing the contact angle value to 0°. The test findings indicate that JFCS achieves a contact angle of 0° at a lower concentration compared to APG, suggesting that JFCS exhibits superior wetting effectiveness in comparison to APG.

**Figure 9:**
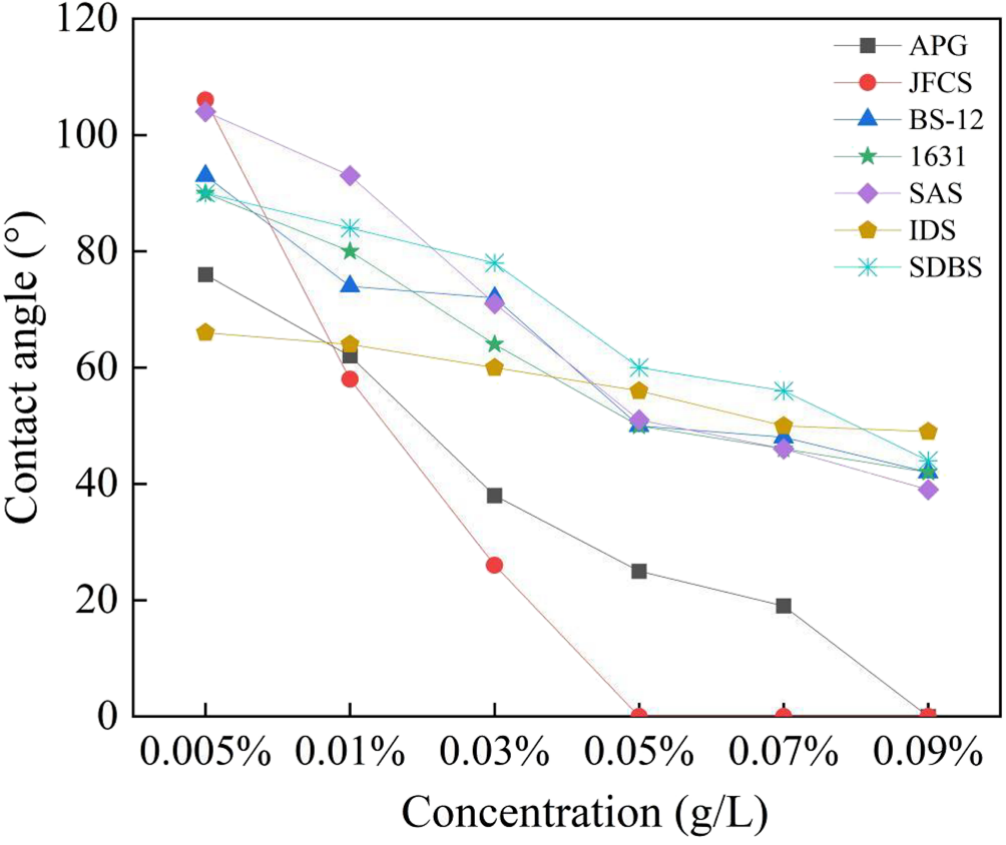
Variation of contact angle with solution mass concentration.

### Surfactant compounding and optimization results

Based on the measurements of surface tension and contact angle, valuable insights can be derived, observably, with the increase in solution concentration, despite the increase in mass concentration, surface tension and contact angle values of IDS are still relatively high and show minimal changes. Therefore, IDS as a coal bed water injection additive, compounding the solution with a surfactant is essential to enhance its wettability. Table 5 shows us the surface tension and contact angle measurements of the complex solution.

**Table 5:** Results of surface tension and contact angle of compounded solutions.

Serial number	Compound solution	Surface tension (mN/m)	Contact angle (°)
M1	SAS + IDS	19.90	55
M2	SDBS + IDS	49.03	65
M3	1631 + IDS	24.70	22
M4	JFCS + IDS	29.70	19
M5	BS-12 + IDS	34.13	64
M6	APG + IDS	28.53	27

(1) Measurement of surface tension for the compounded solution.

**Figure 10:**
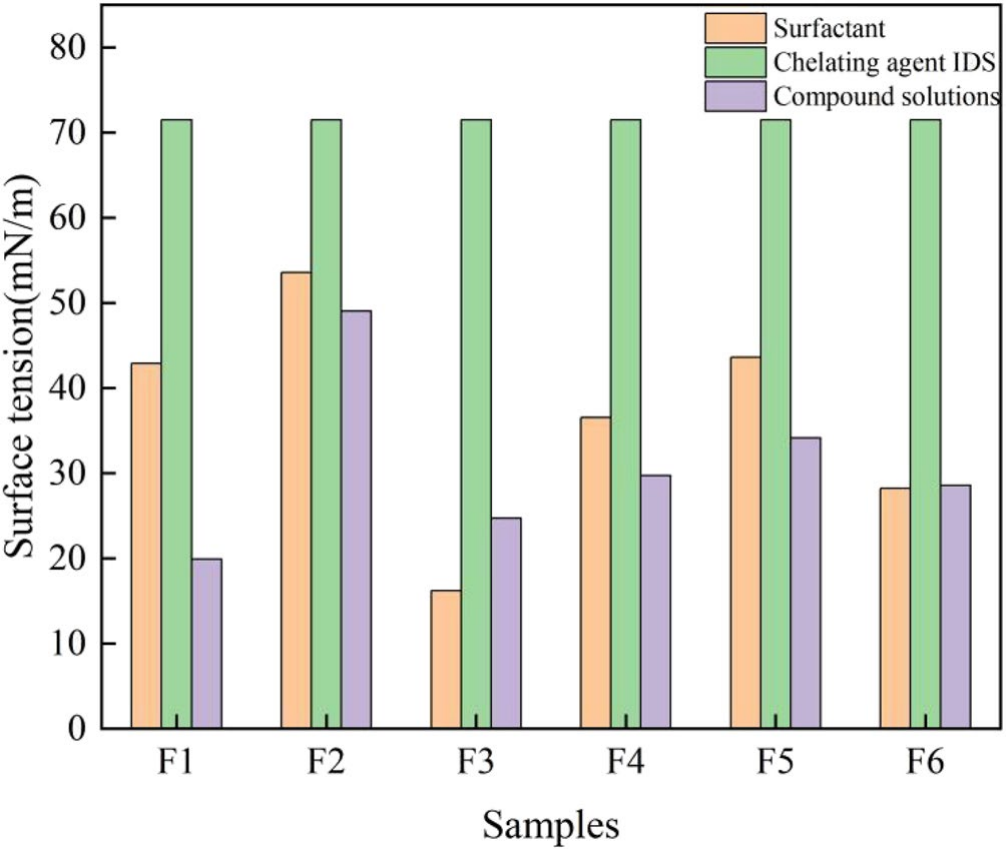
Changes in surface tension of solutions before and after compounding.

Figure 10 depicts the variations in surface tension between the monomer solutions and the compounded solutions. It is evident that the compounded solutions, namely F1, F2, F4, and F5, exhibit lower surface tension compared to their respective monomer solutions. By comparing the surface tension with that of the IDS solution, it becomes apparent that the composite solutions F1, F2, F4, and F5 demonstrate reductions of 31.44%, 72.16%, 58.44%, and 52.25%, respectively. Similarly, when compared to the surface tension of their corresponding surfactant monomer solutions (IDS, surfactant 1631, and APG), the compounded solutions (F1, F2, F4, F5, F3, and F6) exhibit reductions in surface tension by 8.40%, 53.58%, 18.70%, and 21.72%, respectively.Following the compounding process, the composite solution demonstrates a lower surface tension compared to the IDS solution, albeit with a higher surface tension than the monomer surfactant solution. In contrast, the surface tension of the surfactant 1631 solution was 52.75% higher than that of the compounded solution, while the APG solution exhibited a slight increase of 1.06%. Moreover, the chelator IDS solution experienced a significant reduction in surface tension, with reductions of 65.44% and 60.08%, respectively.

(2) Measurement of contact angle for the compounded solution.

**Figure 11:**
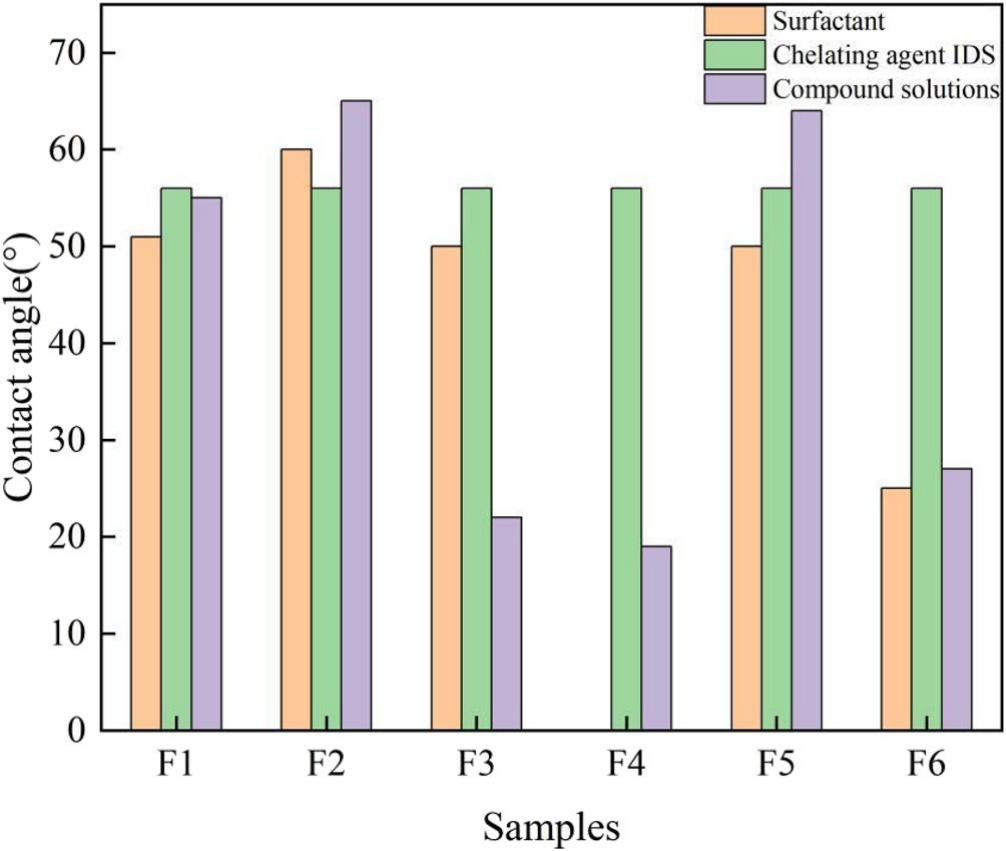
Change in contact angle of solution before and after compounding.

The contact angles of the solutions, both before and after compounding, are presented in Fig. 11. The findings indicate that the contact angle of a composite solution containing IDS and surfactant is significantly smaller than that of the chelator IDS solution. Specifically, the contact angles of complex solutions F1–F6 were reduced by 9.05%, 23.04%, 69.22%, 73.42%, 10.45%, and 62.22%, respectively, compared to the contact angles of IDS solutions. Notably, the contact angle of complex solution F3 was lower than that of both 1631 and IDS solutions when in contact with the coal surface. Specifically, the contact angle of the 1631 solution was reduced by 56%. With the exception of compound solution F3, the contact angles between coal and the other five complex solutions were found to be intermediate between the contact angles of these two types of monomer solutions. Specifically, they all exhibited larger contact angles than the contact angle of the surfactant monomer solution with the coal surface, while displaying lower contact angles than the contact angle of the IDS solution with the coal surface.

**Figure 12:**
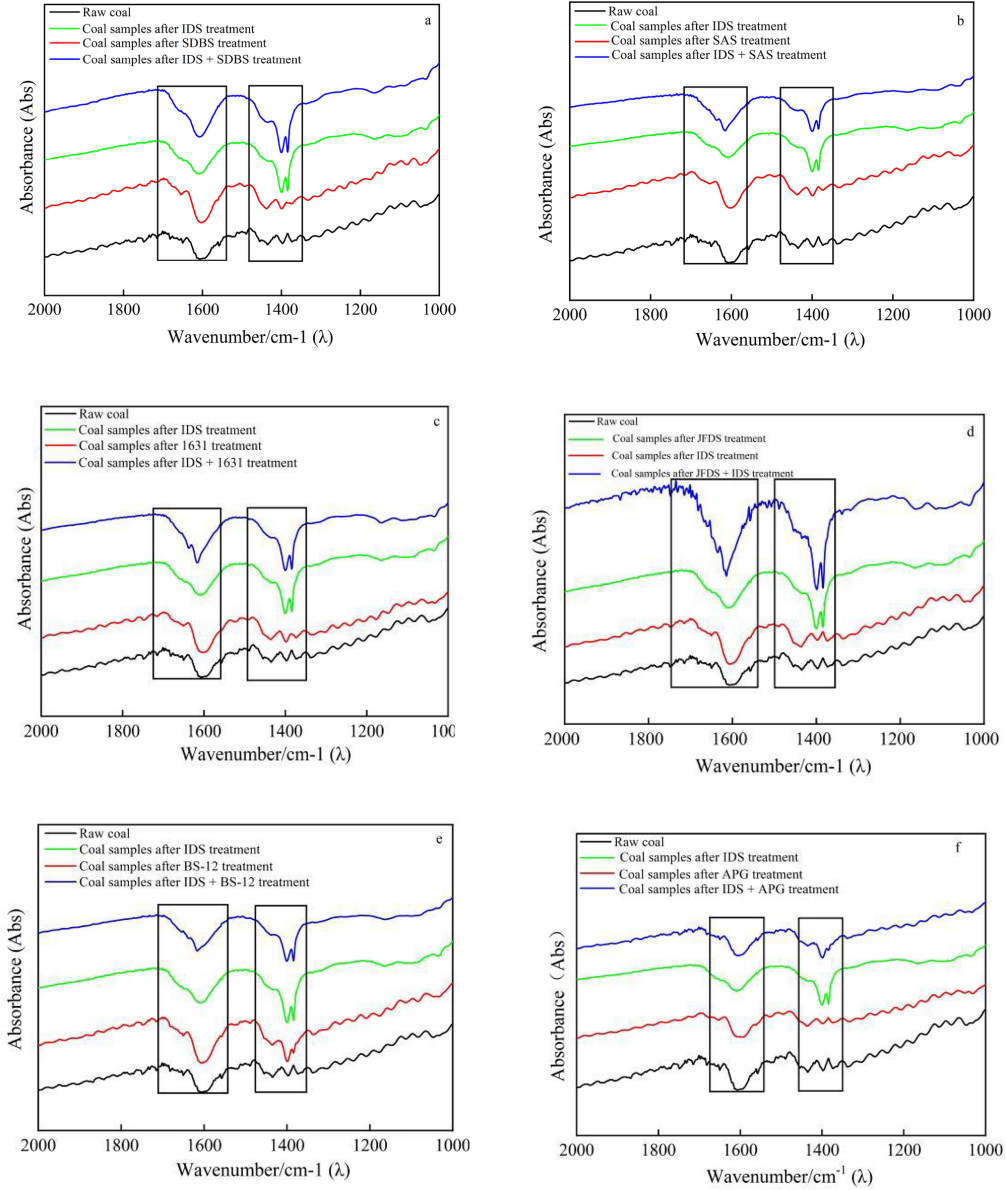
Infrared spectra of coal samples after the action of complex wetting agent.

Figure 12 illustrates the Infrared spectroscopy of coal samples obtained from previous experiments, which were conducted to analyze that the FTIR spectroscopy was employed to analyze the surface functional groups present in coal samples.

Previous research has indicated that the infrared spectra of coal samples reveal absorption bands attributed to oxygen-containing functional groups^19^, primarily within the range of 1200–1800 cm^−1^. Figure 12 illustrates the discernible changes resulting from the application of six different compound solutions. Upon treatment with these compound solutions, a distinct observation in Fig. 12 demonstrates that the coal samples exhibited increased intensity and broader peak shapes within the range of 1718–1555 cm^−1^, accompanied by higher intensity within the range of 1491–1351 cm^−1^. These prominent peaks observed in the coal samples' spectra, spanning from 1718–1555 cm^−1^ to 1491–1351 cm^−1^, correspond to unique vibrations associated with carboxyl (–COOH) and hydroxyl (–OH) groups. It is noteworthy that the primary molecular composition of coal samples predominantly consists of oxygenated functional groups, with a notable presence of –COOH and –OH groups. Figure 11a–f demonstrate that the coal samples treated with the composite solutions exhibited higher intensity and absorption area for the characteristic peaks of –COOH and –OH compared to the two individual monomer solutions. These findings suggest that the composite solution exerts a more pronounced modifying effect on the coal matrix when compared to the two individual monomer solutions. Carboxyl and hydroxyl groups, being highly polar oxygen-containing groups, can establish hydrogen bonds with water through dipole forces. Consequently, this interaction enhances the hydrophilicity and wettability of the coal matrix. The results obtained from the surface tension and contact angle tests conducted using the composite wetting agent and coal samples align with the aforementioned observations.

Based on the experimental findings of surface tension and contact angle presented in Sect. 3.1, notable changes were observed in the compounded solutions resulting from the combination of IDS with surfactants. Despite the combination of IDS with SDBS, the surface tension and contact angle values of the composite solution remained high. Conversely, the compounded solutions of IDS with SAS and BS-12 exhibited reduced surface tension but increased contact angle. These three compounded solutions demonstrated poor wettability performance, particularly at higher concentrations. However, when compounded with 1631, JFCS, and APG at the same concentration, IDS exhibited significantly lower surface tension and contact angle values for all three composite solutions, indicating superior wetting performance.

Among the various compounded solutions applied to the coal samples, the compound solution consisting of IDS and JFCS exhibited the most pronounced characteristic intensity and absorption area of oxygen-containing functional groups on the coal surface, indicating a significant abundance of these functional groups. The alterations in the content of these functional groups were identified as the primary factor influencing the wettability of the coal surface^14^. The higher concentration of oxygen-containing functional groups present on the coal surface signifies improved hydrophilicity and enhanced wetting effects on the coal matrix. Considering the previous experimental findings, as well as economic and environmental considerations, the formulation for the complex wetting agent was determined using the following procedure: the formulation involved the combination of IDS as the chelating agent and JFCS as the surfactant, both with a mass concentration of 0.05%, and a compounding ratio of 1:1.

### An analysis was carried out to assess the influence of the complex wetting agent on coal quality

The coal samples underwent analysis to determine their coal quality, and the results are displayed in Table 6.

**Table 6:** Coal quality measurement results.

Kinds	Dry basis ash (%)	Dry, ash-free volatile matter (%)	Dry basis full sulfur (%)	Air drying base high-level heat generation (MJ/kg)	Coal type
Raw coal	34.36	20.4	4.76	13.1	Coking coal
IDS-soaked coal samples	32.42	19.66	4.12	13.0	Coking coal
Complex wetting agent soaking coal samples	31.22	20.28	4.02	13.3	Coking coal

**Figure 13:**
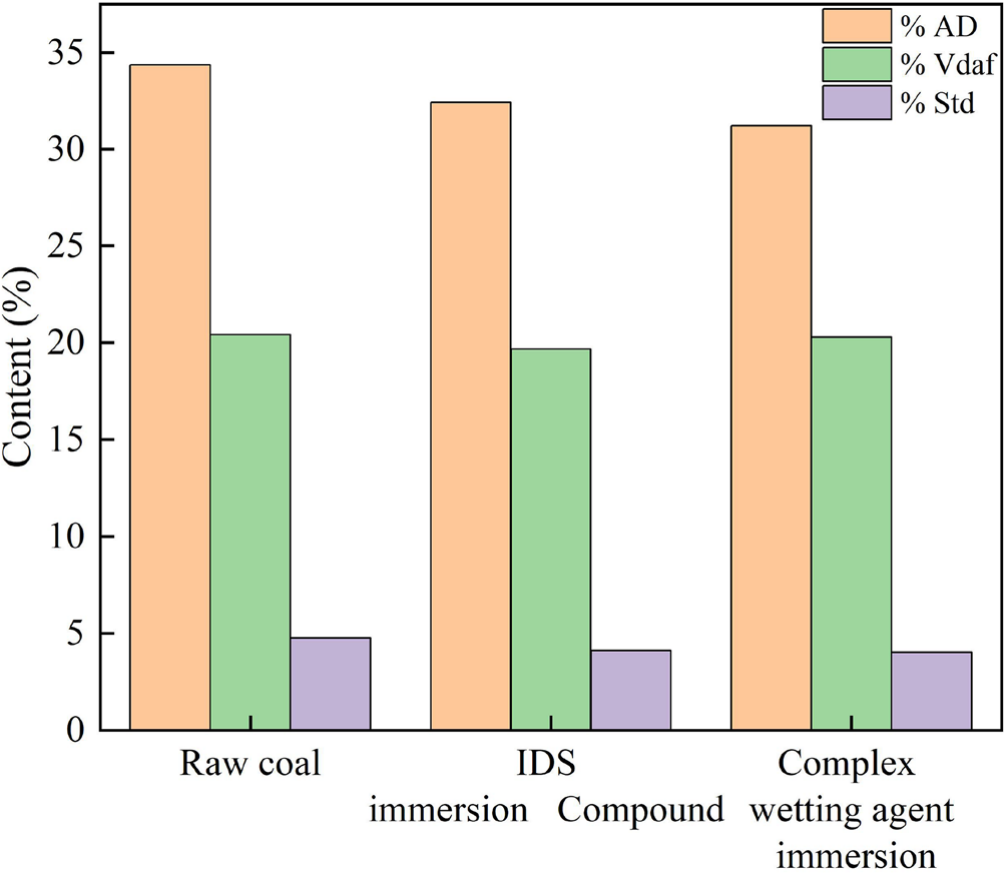
Coal quality changes after the action of complex wetting agent.

Figure 13 depicts the variations in dry basis ash, dry ashless volatile matter, and dry basis total sulfur of the coal following the application of the complex wetting agent.

1. The effect of the complex wetting agent on drying base ash, drying ashless base volatile fraction.
  The dry basis ash content and dry ashless base volatile matter are crucial parameters that influence the calorific value of coal combustion. A lower dry basis ash content corresponds to higher heat generation during coal combustion^20^. Initially, the coal samples exhibited a dry basis ash content of 34.36%. Subsequently, the application of IDS resulted in a decrease in the dry basis ash content to 32.42%, representing a reduction of 5.65%. Furthermore, the utilization of the complex wetting agent further lowered the dry basis ash content to 31.22%, indicating an additional decrease of 9.14%. The reduction in dry basis ash content can be attributed to its primary existence in the form of minerals within the coal. The combined action of the complex wetting agent and IDS promotes the dissolution of minerals, leading to a decrease in the mineral content. Moreover, the improved wettability of coal achieved through JFCS enhances the effectiveness of IDS. Consequently, the dry basis ash content in the coal is reduced, resulting in an increased proportion of combustible components and a higher heating capacity. Conversely, minimal changes were observed in the dry ashless volatile fraction of the coal following the application of IDS and the complex wetting agents. These findings suggest that both IDS and the complex wetting agent have negligible effects on the dry ashless volatile fraction of the coal.
2. The influence of the compound wetting agent on the total sulfur content in the dry basis was examined.
  The sulfur content on a dry basis plays a critical role in assessing the overall quality of coal, as an increase in sulfur content leads to a corresponding rise in the pollutant content of SO_2_ generated during coal combustion. Initially, the coal samples displayed a dry basis total sulfur content of 4.76%. However, upon the application of the chelating agent IDS and the complex wetting agent, the dry basis total sulfur content of the coal decreased to 4.12% and 4.02%, respectively. These reductions represent a decrease of 13.45% and 15.55% compared to the initial coal samples. The complex wetting agent, in conjunction with IDS, facilitates a chelating reaction that promotes the dissolution of pyrite, which is the primary form of inorganic sulfur in coal. As a result, the decrease in the content of inorganic sulfur within the coal leads to a reduction in the total sulfur content on a dry basis. The improved wettability of coal achieved through JFCS enhances the dissolution of pyrite by aiding the action of IDS. Additionally, as the sulfur content in coal diminishes, the subsequent combustion process generates lower levels of SO_2_ pollutants. Consequently, the reduction in sulfur content not only contributes to decreased production of SO_2_ pollutants during combustion but also results in reduced environmental pollution in the atmosphere.

### Analysis of the influence of composite wetting agent on the water content of coal body

The water content measurements of coal samples following immersion in various solutions are presented in Table 7.

**Table 7:** Coal body water content measurement results.

Type of coal sample	Serial number	Mass of coal samples (g)	Quality of dried coal samples (g)	Moisture content (%)	Average moisture content (%)
Raw coal	1	323.97	288.23	11.03%	
	2	315.36	279.85	11.26%	10.56
	3	318.79	288.87	9.39%	
Water-soaked coal samples	1	335.53	279.22	16.78%	
	2	310.73	264.82	14.78%	15.78
	3	322.32	271.49	15.77%	
IDS-soaked coal samples	1	318.95	260.43	18.35%	
	2	330.12	268.79	18.58%	18.88
	3	320.76	257.55	19.71%	
JFCS immersion coal sample	1	312.74	255.11	18.43%	
	2	320.58	253.75	20.85%	19.85
	3	329.68	262.86	20.27%	
Compound wetting agent soaking coal samples	1	330.59	259.78	21.42%	
	2	326.32	258.25	20.86%	20.86
	3	323.11	257.51	20.30%	

**Figure 14:**
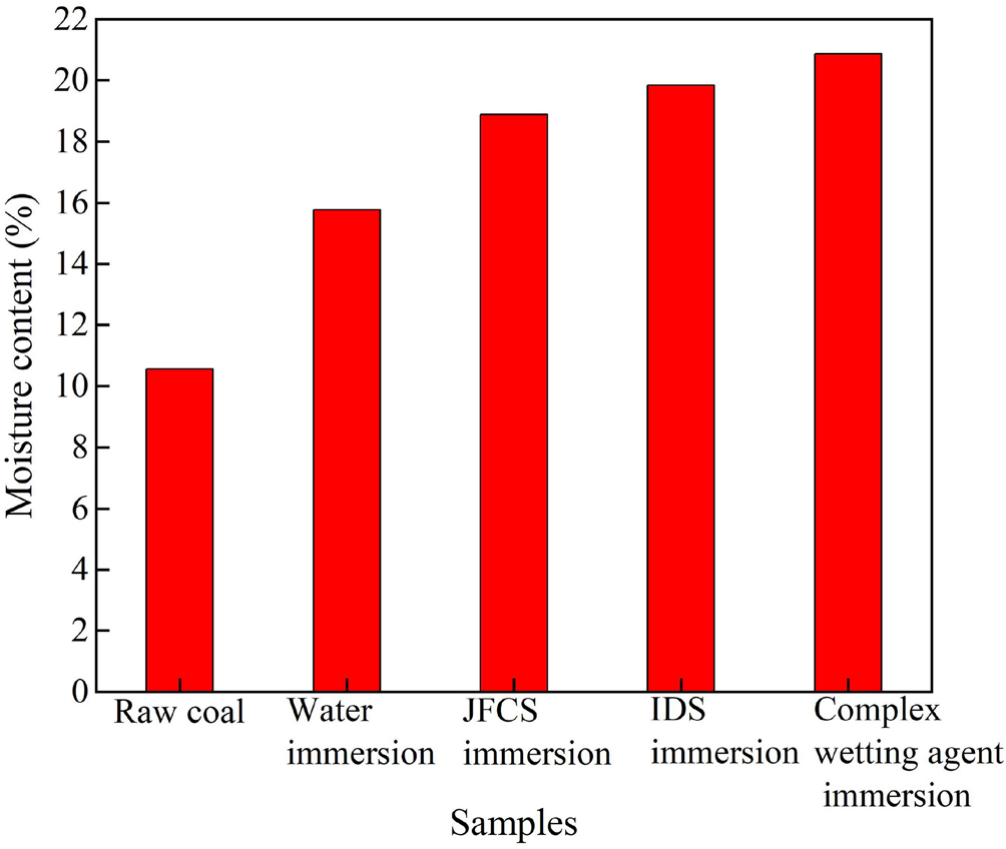
Changes in average moisture content of coal samples after the action of complex wetting agents.

Figure 14 illustrates the variations in the mean moisture content of the coal samples after the application of the complex wetting agent.

The figure presented illustrates an increase in the moisture content of the coal samples subsequent to the application of various solutions, with the complex wetting agent demonstrating the most significant elevation. Immersion in ultrapure water leads to an increase in the initial moisture content of 10.56–15.78%, representing a relative increase of 1.49 times compared to the original coal. The utilization of JFCS results in a subsequent rise in the moisture content of the coal samples to 18.88%, indicating a relative growth of 8.32% compared to the original moisture level. Similarly, the application of IDS leads to an increase in the water content of the coal sample to 19.85%, signifying a 9.29% relative increase compared to the initial moisture level of the coal. Remarkably, the application of the compound wetting agent yields the highest increase in water content, reaching 20.86%. This represents a substantial elevation compared to the initial water content and a 9.29% increase compared to the original coal. Moreover, the moisture content of the coal sample exhibits a significant augmentation following the implementation of the complex wetting agent, which is 1.98 times that of the raw coal and 1.32 times that of the ultrapure water-treated coal sample. These findings demonstrate the substantial enhancement in water absorption achieved through the utilization of complex wetting agents.

**Figure 15:**
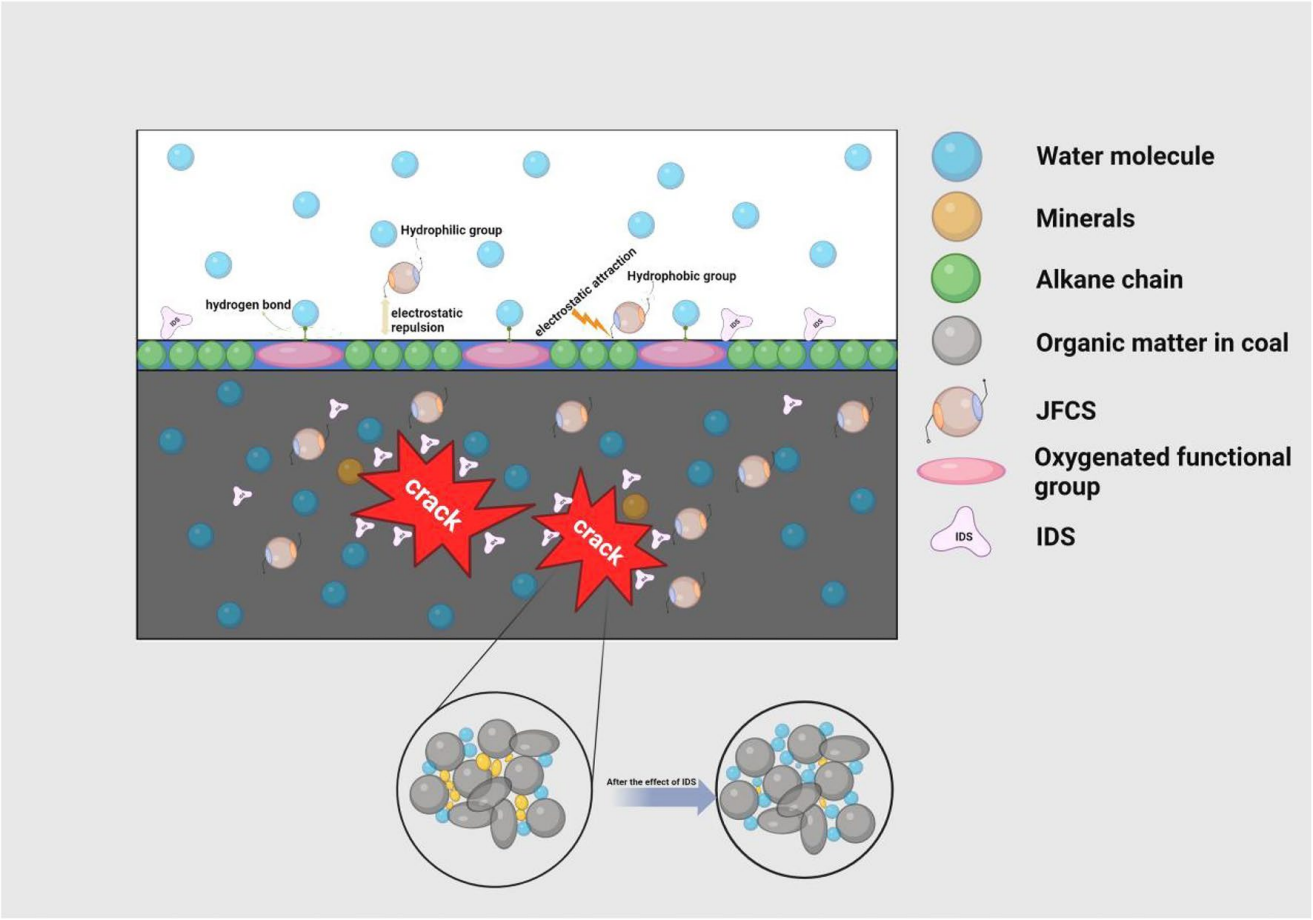
Complex wetting agent mechanism.

The presence of pores and cracks within the coal matrix facilitates the infiltration of ultrapure water into its interior, resulting in an expansion of the wetting area and a subsequent increase in the coal's water content^21^. The hydrophobic nature of the coal surface, attributed to the presence of hydrophobic groups such as aliphatic and aromatic hydrocarbons, impedes the wetting ability of water on the coal surface^21^. However, the introduction of JFCS into the water leads to its adsorption onto the coal surface. The hydrophilic groups present in JFCS extend towards the solution, enhancing the hydrophilicity of the coal surface. This hydrophilic modification ultimately leads to an increase in the water content within the coal matrix. The application of IDS results in the dissolution of mineral particles within the coal, thereby increasing its porosity. This augmented porosity enhances the wetting effect of the coal and subsequently elevates its water content^22^. Moreover, the introduction of the complex wetting agent induces the opening of secondary pores within the coal matrix, further increasing its porosity. Additionally, the complex wetting agent enhances the hydrophilicity of the coal surface, leading to a significant improvement in both the wetting effect and water content of the coal. The water content of the coal directly influences the effectiveness of wetting the coal matrix, as indicated by Ref.^23^. In summary, an increased water content in the coal corresponds to a more favorable wetting effect on the coal matrix. Figure 15 illustrates the mechanism by which the complex wetting agent operates.

### Evaluation of the impact of composite wetting agents regarding the physical properties of coal samples.

#### The influence of the composite wetting agent on the uniaxial compressive strength (*σ*_*c*_)

**Figure 16:**
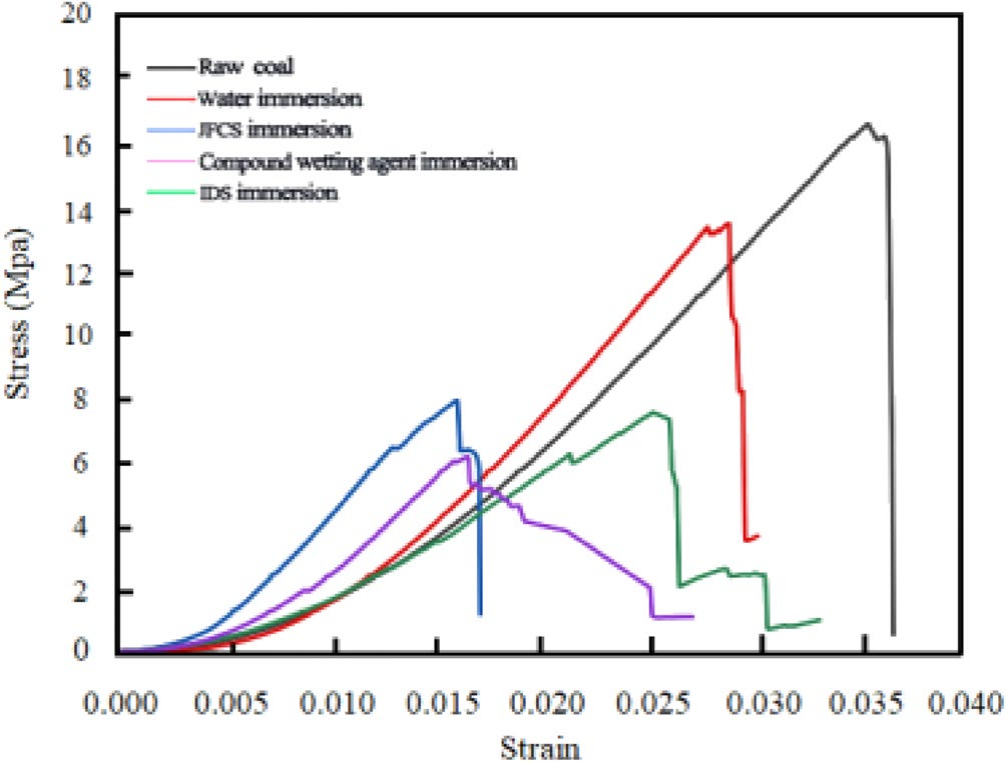
Stress–strain after uniaxial compression of different coal samples.

The stress–strain diagrams, load–displacement diagrams, and load-time diagrams obtained by analyzing the uniaxial compression process are shown in Figs. 16, 18, and 20.

Figure 16 shows the stress–strain variation of coal samples during uniaxial compression.

Figure 16 shows the load–displacement variations of the coal samples during uniaxial compression. The uniaxial compressive strength *σ*_*c*_ of the coal samples was determined. What’s more, the magnitude of the *σ*_*c*_ value can be used to assess the propensity of the coal body to experience impact hazards^24^. Figure 16 illustrates the change in the *σ*_*c*_ of coal samples after the application of a complex wetting agent.

**Figure 17:**
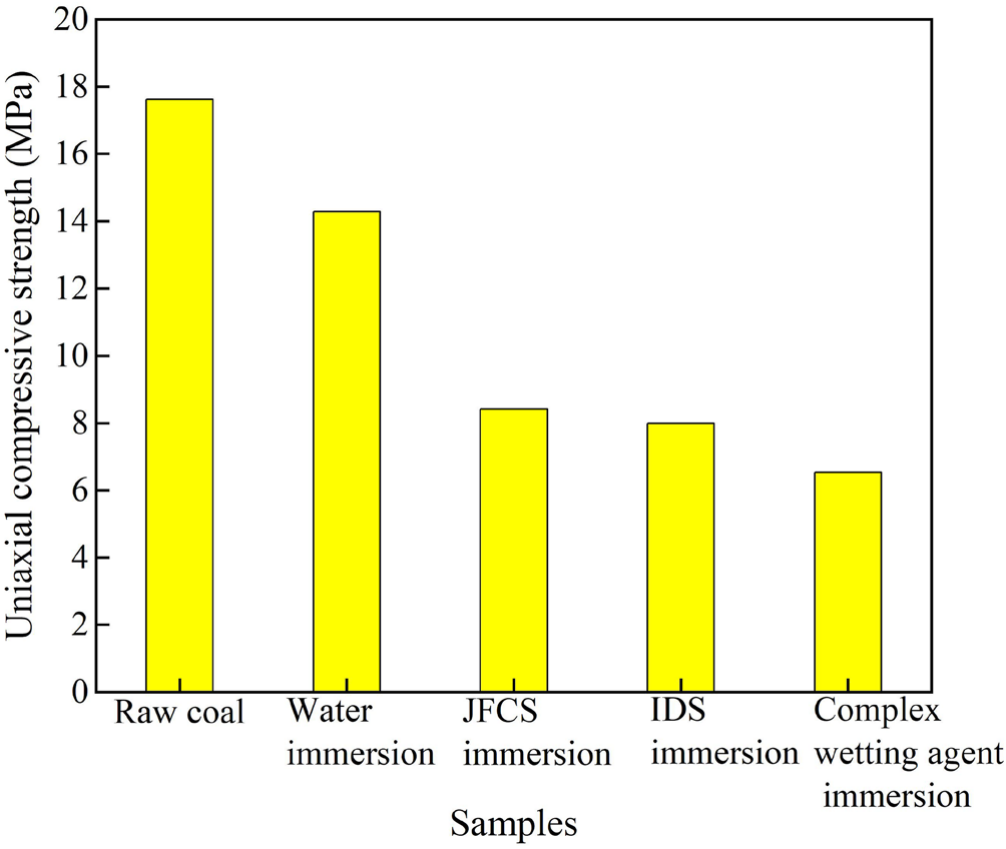
Changes in uniaxial compressive strength of coal samples after the action of complex wetting agents.

Figure 17 depicts that the original coal sample possesses a *σ*_*c*_ value of 17.62 MPa. Upon exposure to ultrapure water, the *σ*_*c*_ value of the coal sample decreases to 14.29 MPa, signifying an 18.88% reduction. Furthermore, the implementation of JFCS leads to a decrease in the *σ*_*c*_ value of the coal samples to 8.41 MPa, resulting in a reduction of 9.21 MPa compared to the original coal sample. Subsequently, the application of IDS causes the *σ*_*c*_ value of the coal sample to decrease to 8.00 MPa, indicating a substantial reduction of 54.61%. Similarly, the utilization of the complex wetting agent also yields a *σ*_*c*_ value of 8.00 MPa, with an equivalent reduction of 54.61%. Notably, the *σ*_*c*_ value of the coal sample experiences a further decrease to 6.53 MPa after the action of the complex wetting agent, representing a remarkable reduction of 62.93% compared to the original coal sample. This reduction is significantly greater than that observed in coal samples treated with alternative solutions.

#### Effect of composite wetting agents on the impact energy index (***K***_***E***_)

**Figure 18:**
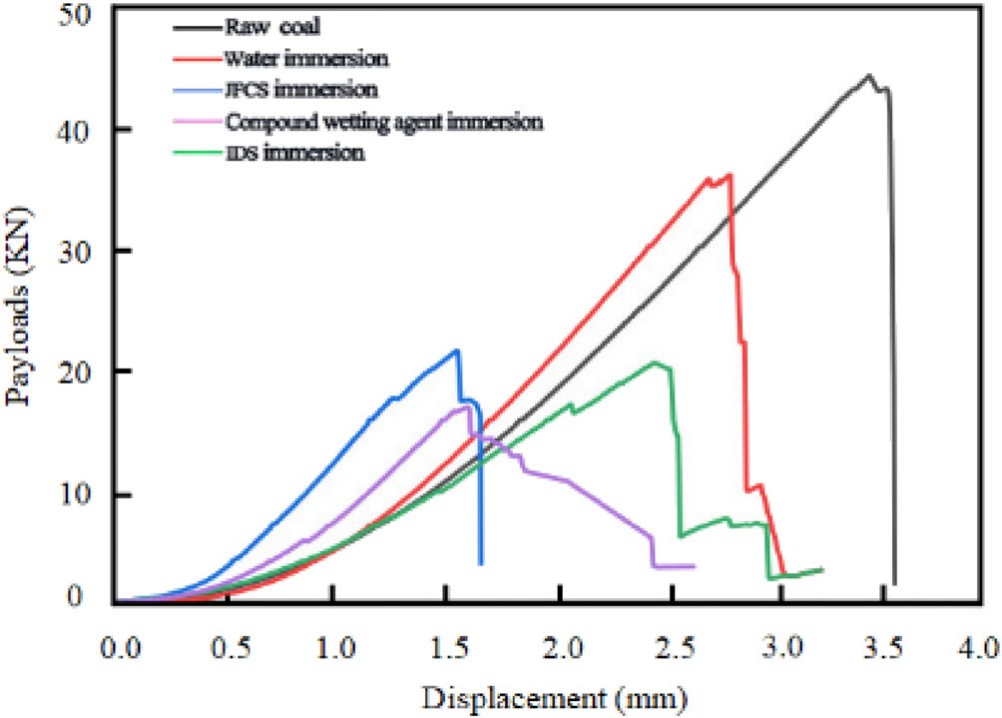
Load displacement variation of different coal samples after uniaxial compression.

Figure 18 shows the load–displacement variations of the coal samples during uniaxial compression.

**Figure 19:**
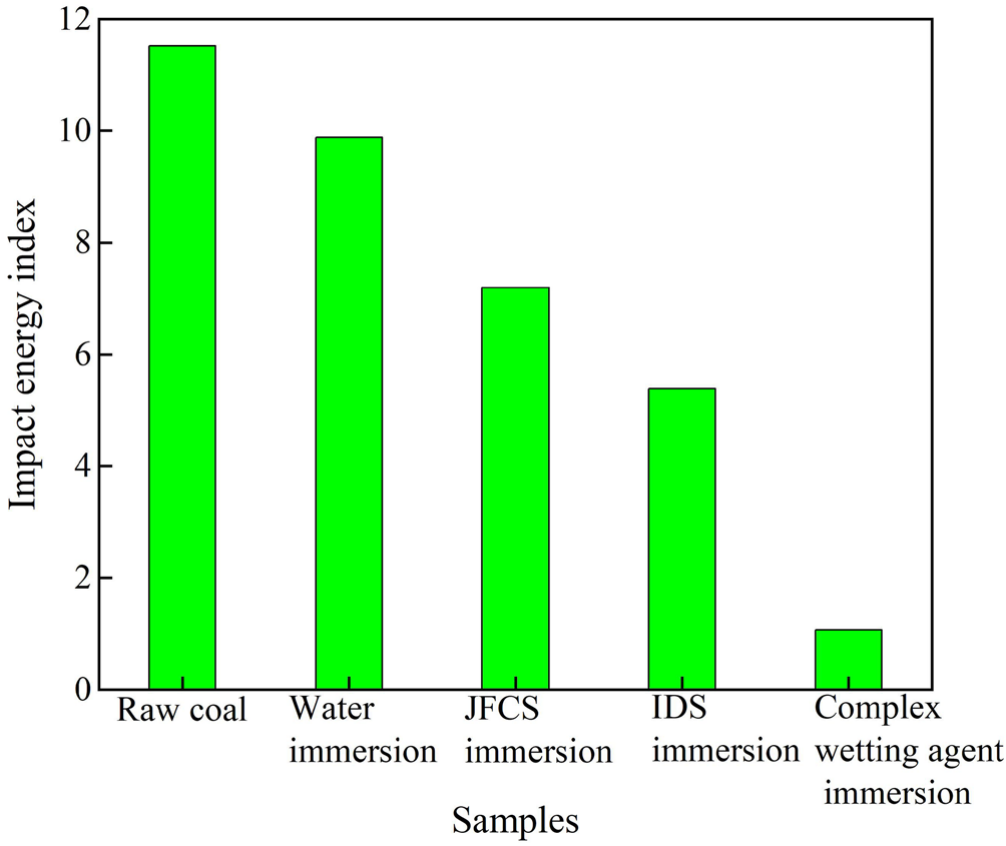
Variation of impact energy index of coal samples after the action of complex wetting agents.

The impact propensity of coal samples is inversely related to the *K*_*E*_. In other words, coal samples with lower *K*_*E*_ values exhibit a reduced tendency for impact^25^. Figure 19 shows us the change in the* K*_*E*_ value of the coal samples after using the complex wetting agent.

Figure 19 illustrates that the original coal sample exhibits a higher *K*_*E*_ value of 11.52. Following the application of ultrapure water, the *K*_*E*_ value decreases to 9.88, indicating a reduction of 14.28%. Subsequently, the utilization of JFCS leads to a decrease in the *K*_*E*_ value of the coal sample by 4.33, resulting in a value of 7.19 compared to the original coal sample, and a reduction of 2.68 compared to the ultrapure water treatment. Moreover, the implementation of IDS causes the *K*_*E*_ value to decrease to 5.39, representing a reduction of 6.13% compared to the original coal sample. Remarkably, the coal samples treated with the complex wetting agent exhibit the most substantial reduction in the *K*_*E*_ value. The *K*_*E*_ value decreases from 11.52 to 1.07, resulting in a remarkable reduction of 90.74%. Through the experimental findings, it is evident that the *K*_*E*_ value is reduced by 6.35 times compared to the coal samples treated with ultrapure water, 2.42 times compared to the coal samples treated with JFCS, and 1.71 times compared to the coal samples treated with IDS.

#### Effect of complex wetting agents on dynamic destruction time (***D***_***T***_)

**Figure 20:**
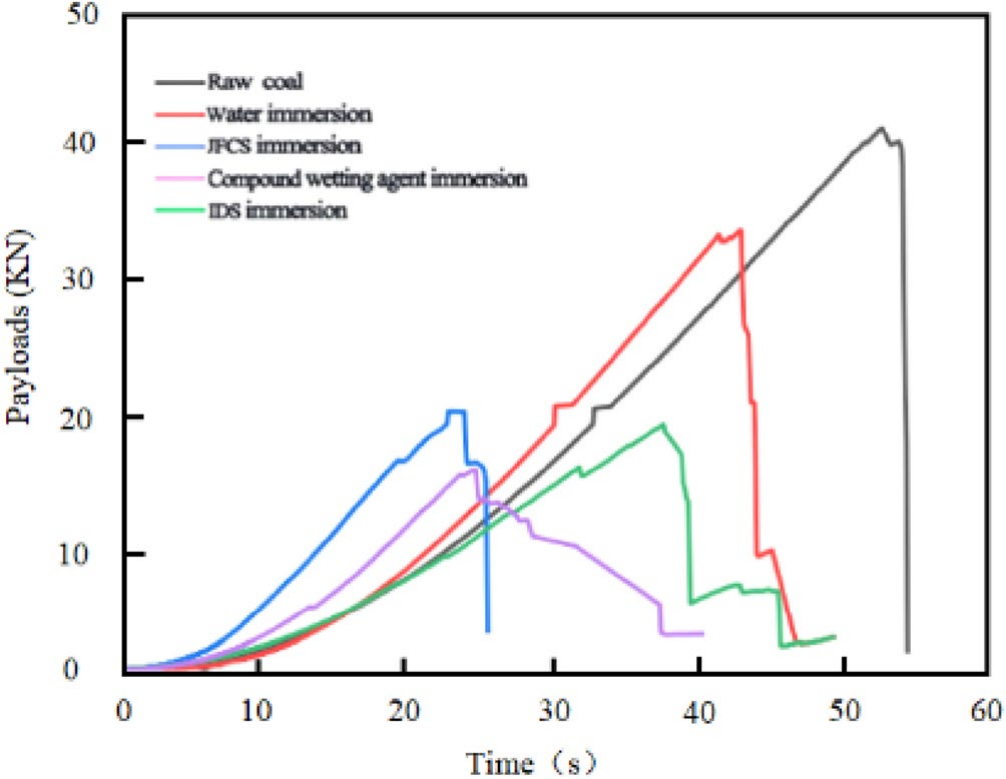
Load time variation of different coal samples after uniaxial compression process.

Figure 20 shows the load-time variation during uniaxial compression of a coal sample.

Dynamic destruction time *D*_*T*_ is used as one of the determination indexes of coal rock impact propensity, as the value of *D*_*T*_ increases, the impact propensity of coal samples is decreased^26^. Figure 20 shows the variation of *D*_*T*_ of coal samples after using complex wetting agents.

**Figure 21:**
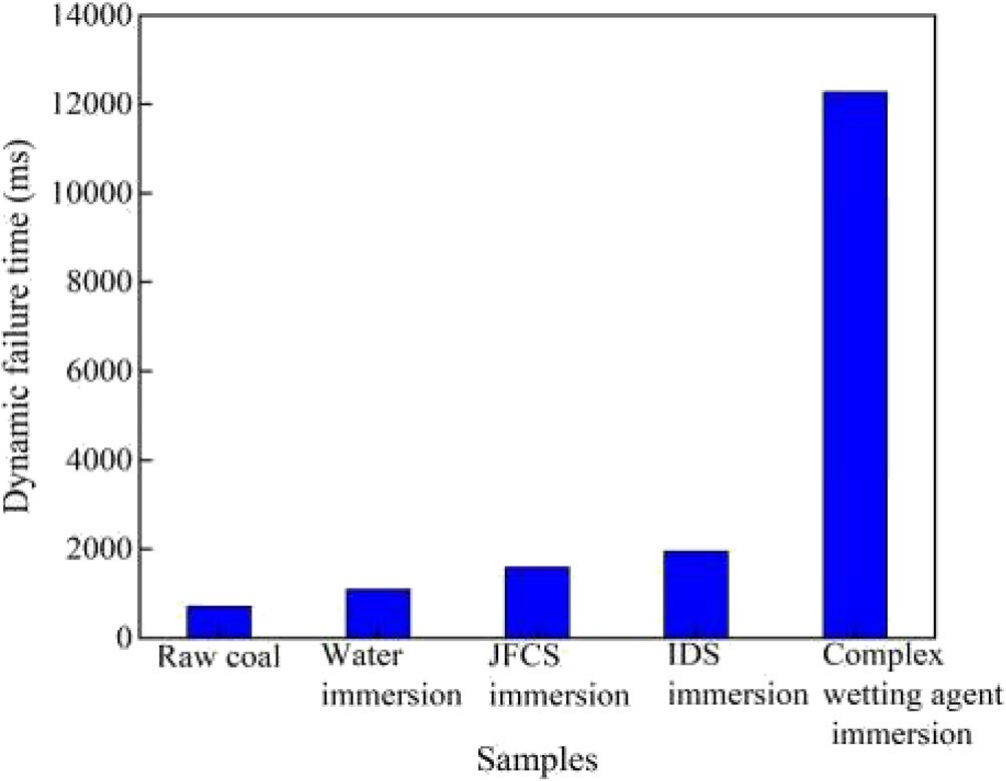
Dynamic damage time changes in coal samples after the action of complex wetting agents.

Upon analyzing Fig. 21, it becomes evident that the utilization of different solutions results in an augmentation of the *D*_*T*_ value in the coal samples. The initial coal sample exhibited a* D*_*T*_ value of 711.3 ms. Following the application of ultrapure water, the *D*_*T*_ value of the coal sample increased to 1094.39 ms, representing a dynamic destructive time increment of 383.09 ms. Subsequently, the implementation of JFCS led to a substantial enhancement in the *D*_*T*_ value of the coal samples, reaching 1586.87 ms. This value surpassed the original coal samples by 875.57 ms, and the *D*_*T*_ value of the JFCS-treated coal samples was 2.23 times greater than that of the ultrapure water-treated coal samples. Furthermore, the application of IDS resulted in a* D*_*T*_ value of 1942.54 ms for the coal sample post-treatment, demonstrating a significant increase of 2.73 times compared to the original coal sample.

It is noteworthy that among all the treated coal samples, the coal sample subjected to the complex wetting agent exhibited the most substantial enhancement in the *D*_*T*_ value. With the utilization of the complex wetting agent, the *D*_*T*_ value of the coal samples remarkably increased to 12,270.9 ms, representing an astonishing 17.25-fold augmentation compared to the original coal sample. Furthermore, the* D*_*T*_ value of the coal sample treated with the complex wetting agent exhibited a remarkable increase by a factor of 11.21 compared to coal samples treated with ultrapure water, 7.73 times compared to coal samples treated with JFCS, and an impressive 6.32 times compared to coal samples treated with IDS.

#### Analysis of the causes of changes in the physical properties of coals

According to the analysis of the *σ*_*c*_ of coal samples, Table 8 shows us the results of the measurements concerning the *K*_*E*_ and the *D*_*T*_ after the application of the complex wetting agent, along with an evaluation of the impact propensity of the coal samples.

**Table 8:** Determination of the impact tendency of coal samples after the action of different solutions.

Type of coal sample	Dynamic destruction time (ms)	Impact energy index	Uniaxial compressive strength (Mpa)
Raw coal	None	Vigorous	Vigorous
Water-soaked coal samples	None	Vigorous	Vigorous
IDS-soaked coal samples	None	Vigorous	Weak
JFCS immersion coal sample	None	Vigorous	Weak
Complex wetting agent soaking coal samples	None	None	None

**Figure 22:**
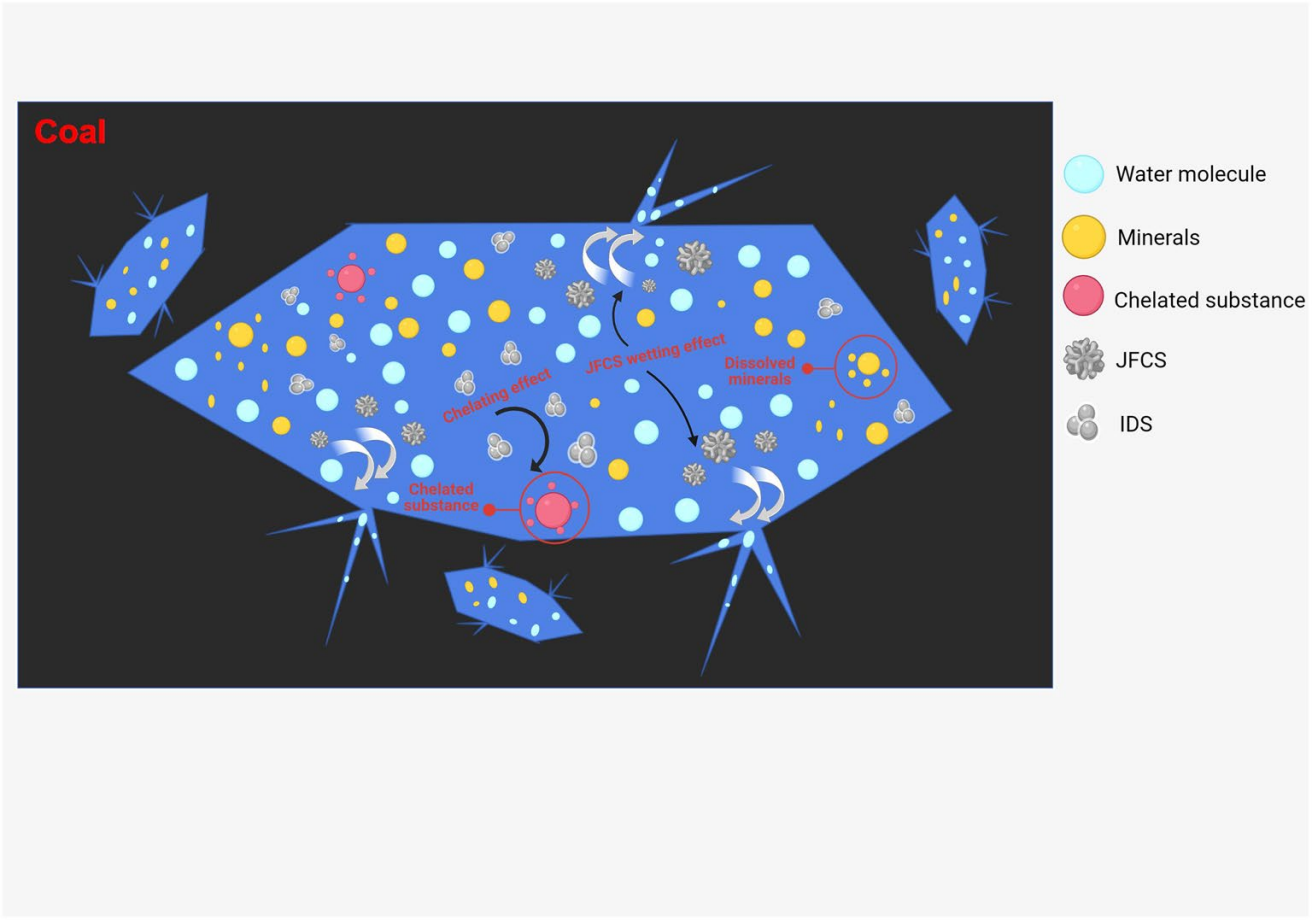
Mechanism of change in coal physical properties after the action of complex wetting agent.

The modification of the mechanical properties of the coal body can be attributed to several key factors. Firstly, the infiltration of ultrapure water into the pores of the coal body and its subsequent adsorption result in an increased water content, thereby weakening the intermolecular connections^27^. Secondly, the surfactant JFCS reduces the surface tension of the solution, thereby altering the characteristics of the interface between the two phases and increasing the moisture content within the coal body. This enhances the mobility of water within the internal pores of the coal samples, leading to a reduction in strength and an increase in plasticity of the coal body^28^. Furthermore, the chelating agent IDS interacts with the mineral particles and cement within the coal, causing their dissolution and the formation of new fissures. Consequently, the pre-existing internal cracks in the coal samples become interconnected, forming a network-like structure^29,30^. The combined influence of IDS and JFCS brings about changes in the mechanical characteristics and internal composition of the coal samples. This process leads to the development and interconnection of cracks within the coal body, further enhancing the network-like structure. As a result, the water content within the cracks increases, thereby improving the mobility of water within them. Additionally, during the destruction process, the moisturization of the coal body is significantly improved. This prevents the accumulation of excessive elastic energy within the coal body, leading to its destabilization primarily through viscous destruction. Consequently, the *K*_*E*_ and *σ*_*c*_ values of the coal samples are reduced, resulting in an increase in the *D*_*T*_ value and a decrease in the propensity of the coal to undergo impact-induced damage. Figure 22 illustrates the mechanism by which the physical properties of the coal body change when a complex wetting agent is utilized.

## Conclusion

The surface tension and contact angle of the seven monomer solutions exhibited a gradual decrease as the mass concentration increased. However, the contact angle between the chelator IDS solution and the coal surface showed minimal variation with increasing mass concentration, indicating poor wettability. To enhance the solution's wettability, it was necessary to combine it with a surfactant. Experimental investigations were conducted to determine the surface tension, contact angle, and content of oxygen-containing functional groups on the coal surface of the coal samples. Subsequently, the optimal formulation and concentration of the complex wetting agent were established: a mass concentration of 0.05% for both the chelating agent IDS and the surfactant JFCS. The compounding ratio was determined to be 1:1.

After the application of the complex wetting agent, the observed outcomes and their impact were evaluated. The coal's dry basis ash and dry basis full sulfur were reduced by 9.14% and 13.45% respectively, resulting in a decrease in the ash content of the coal. The utilization of the complex wetting agent led to an increase in the combustible component content, thereby enhancing the coal's heating capacity. Additionally, the application of the wetting agent resulted in a decrease in the inorganic sulfur content of the coal, leading to lower production of SO_2_ pollutants during combustion and reduced pollution of the atmospheric environment. Furthermore, the moisture content of the coal samples increased to 20.86%, which was 1.98 times higher than the original coal. Moreover, the* K*_*E*_ value of the coal body exhibited a significant decline of up to 90.74% compared to the original coal. Additionally, the σc value of the coal decreased by 62.93% compared to the original coal, indicating a substantial reduction in strength. The *D*_*T*_ value increased to 12,270.9 ms, which was 17.25 times higher than that of the raw coal.

The incorporation of the complex moistening agent resulted in significant modifications to the quality, physical and mechanical characteristics, as well as the internal structure of the raw coal. The chelating agent IDS played a crucial role in the development and interconnection of cracks within the coal body, leading to the formation of a network that enhanced the coal's plasticity. On the other hand, the surfactant JFCS significantly reduced the surface tension of the solution, thereby altering the properties of the two-phase interface. Consequently, this increased the mobility of water within the internal pores of the coal sample, thereby improving its wetting ability. The combined effect of JFCS and IDS facilitated the formation and interconnection of fissures within the coal body, creating a network-like structure. This, in turn, increased the water content within the fissures and improved the fluidity of water within them. As a result, the permeability of the coal body was enhanced, and its susceptibility to damage caused by impact was reduced. These findings offer a novel perspective for the prevention and control of impact-induced pressure in deep coal mining.

## Data Availability

The data used to support the findings of this study are available from the first author upon request.
